# Supplementary material for: The chromatin, topological and regulatory properties of pluripotency-associated poised enhancers are conserved in vivo
Source: Nat Commun. 2021 Jul 16;12:4344. doi: 10.1038/s41467-021-24641-4 (PMC8285398; doi:10.1038/s41467-021-24641-4)
Supplement: Supplementary file 1 — Supplementary Information [file 41467_2021_24641_MOESM1_ESM.pdf]

Supplementary Figures

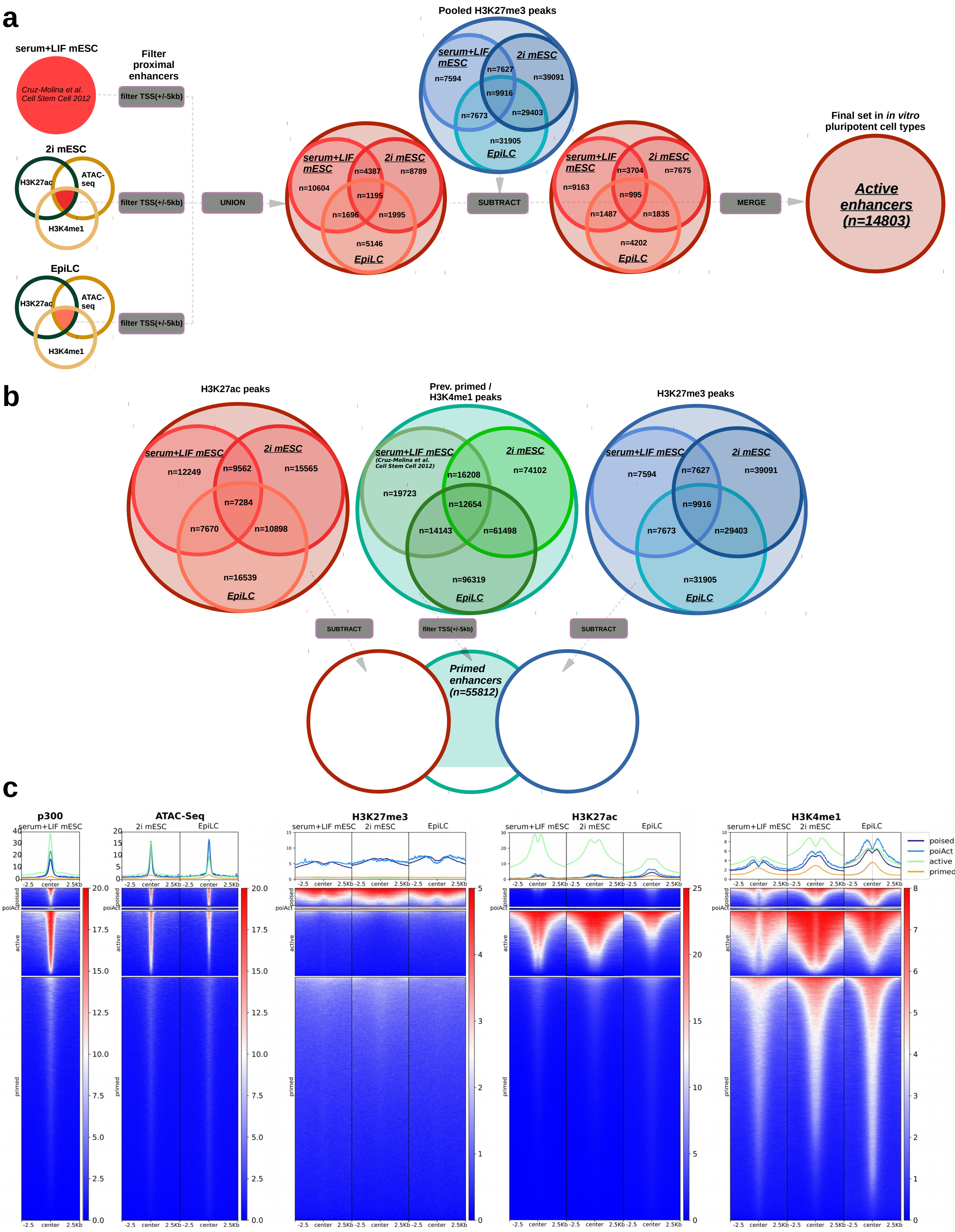

Supplementary Figure 1: Epigenomic features of PEs during mouse embryogenesis. (A) ATAC-seq and ChIP-seq data for p300, H3K4me1, H3K27me3 and H3K27ac were used to call active enhancers in three distinct *in vitro* pluripotent states (i.e. 2i ESC, S+L ESC and EpiLC)[5,80]. Active enhancers are characterized by high p300/ATAC signals, high H3K27ac and H3K4me1 levels, as well as low H3K27me3 levels and located at least 5kb apart from a TSS (**Methods**). **(B)** ChIP-seq data for H3K4me1, H3K27me3 and H3K27ac were used to call primed enhancers in three distinct *in vitro* pluripotent states (i.e. 2i ESC, S+L ESC and EpiLC)[5,80]. Primed enhancers are characterized by high H3K4me1 signals, low H3K27ac levels and H3K27me3 levels and located at least 5kb apart from a TSS (**Methods**). **(C)** H3K27ac, H3K4me1 and H3K27me3 signals from serum+LIF mESC, 2i mESC and EpiLC[1] are shown around poised, active, primed and PoiAct enhancers identified in *in vitro* pluripotent cells (**Fig. 1; Methods**).

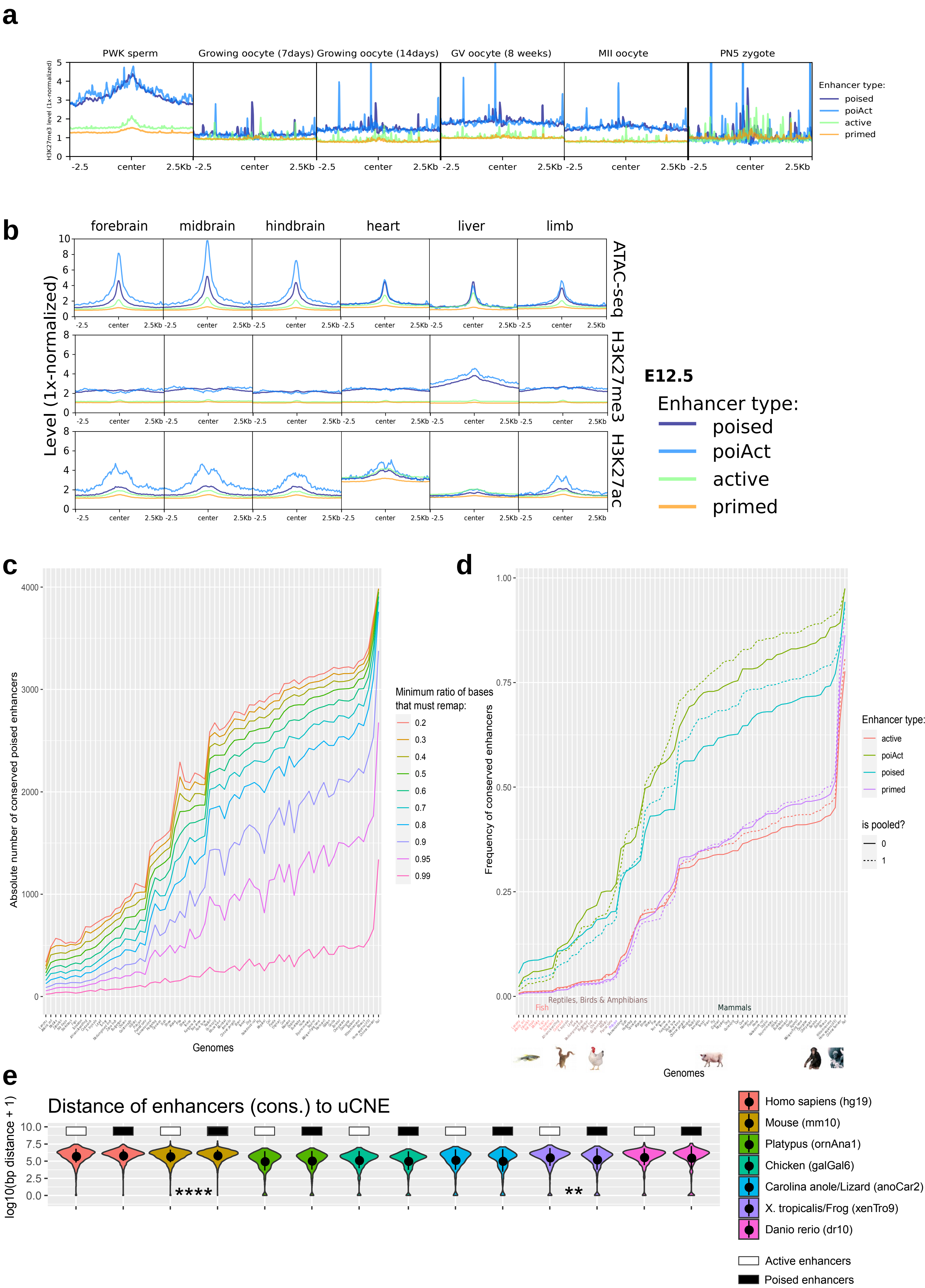
a
PWK sperm
Growing oocyte (7days)
Growing oocyte (14days)
GV oocyte (8 weeks)
MII oocyte
PN5 zygote
H3K27me3 level (1x-normalized)
-2.5
center
2.5Kb
Enhancer type:
poised
poiAct
active
primed
b
forebrain
midbrain
hindbrain
heart
liver
limb
Level (1x-normalized)
ATAC-seq
H3K27me3
H3K27ac
E12.5
Enhancer type:
poised
poiAct
active
primed
c
Absolute number of conserved poised enhancers
Genomes
Minimum ratio of bases that must remap:
0.2
0.3
0.4
0.5
0.6
0.7
0.8
0.9
0.95
0.99
d
Frequency of conserved enhancers
Fish
Reptiles, Birds & Amphibians
Mammals
Genomes
Enhancer type:
active
poiAct
poised
primed
is pooled?
0
1
e
Distance of enhancers (cons.) to uCNE
log10(bp distance + 1)
****
**
Homo sapiens (hg19)
Mouse (mm10)
Platypus (ornAna1)
Chicken (galGal6)
Carolina anole/Lizard (anoCar2)
X. tropicalis/Frog (xenTro9)
Danio rerio (dr10)
Active enhancers
Poised enhancers

Supplementary Figure 2: Conservation analysis of mouse PE across vertebrates. (A) H3K27me3 ChIP-Seq profiles generated in male and female mouse germ cells[2] are shown around poised, active, primed and PoiAct enhancers identified in *in vitro* pluripotent cells. **(B)** ATAC-seq, H3K27me3 and H3K27ac signals from six different mouse embryonic (E12.5; upper panel) tissues are shown around poised, active, primed and PoiAct enhancers identified in *in vitro* pluripotent cells. **(C)** Absolute number of mouse PEs conserved across 68 vertebrates (**Supplementary Data 1**) using different mappability thresholds (mapping ratios 0.2-0.99; color legend)**. (D)** Sequence conservation across 68 vertebrate species was measured for poised, active, primed and PoiAct enhancers identified in mouse pluripotent cells using a mappability threhold of 0.5. The previous enhancer sets were further divided depending on whether they were called considering several pluripotent cell types as defined in **Fig. 1** („pooled"; solid lines) or only in serum+LIF mESC as originally described[3] (dashed lines). **(E)** Distance between active or poised mouse enhancers conserved in the indicated vertebrate species and ultraconserved non-coding elements (uCNE) identified in the same species[4]. The asterisks indicate that the distances between either poised or active enhancers and uCNU are significantly different (two-sided Wilcoxon test). Human and frog PE (FC=1.16, p=0.052; FC=1.33, p=0.0034; two-sided Wilcoxon test) are closer to uCNE, but mouse and zebrafish PE (FC=0.86, p=4.08e-12; FC=0.68, p=0.31; two-sided Wilcoxon test) are more distal to uCNE when compared to active conserved enhancers. * $p \leq 0.05$, ** $p \leq 0.01$, *** $p \leq 0.001$, **** $p \leq 0.0001$.

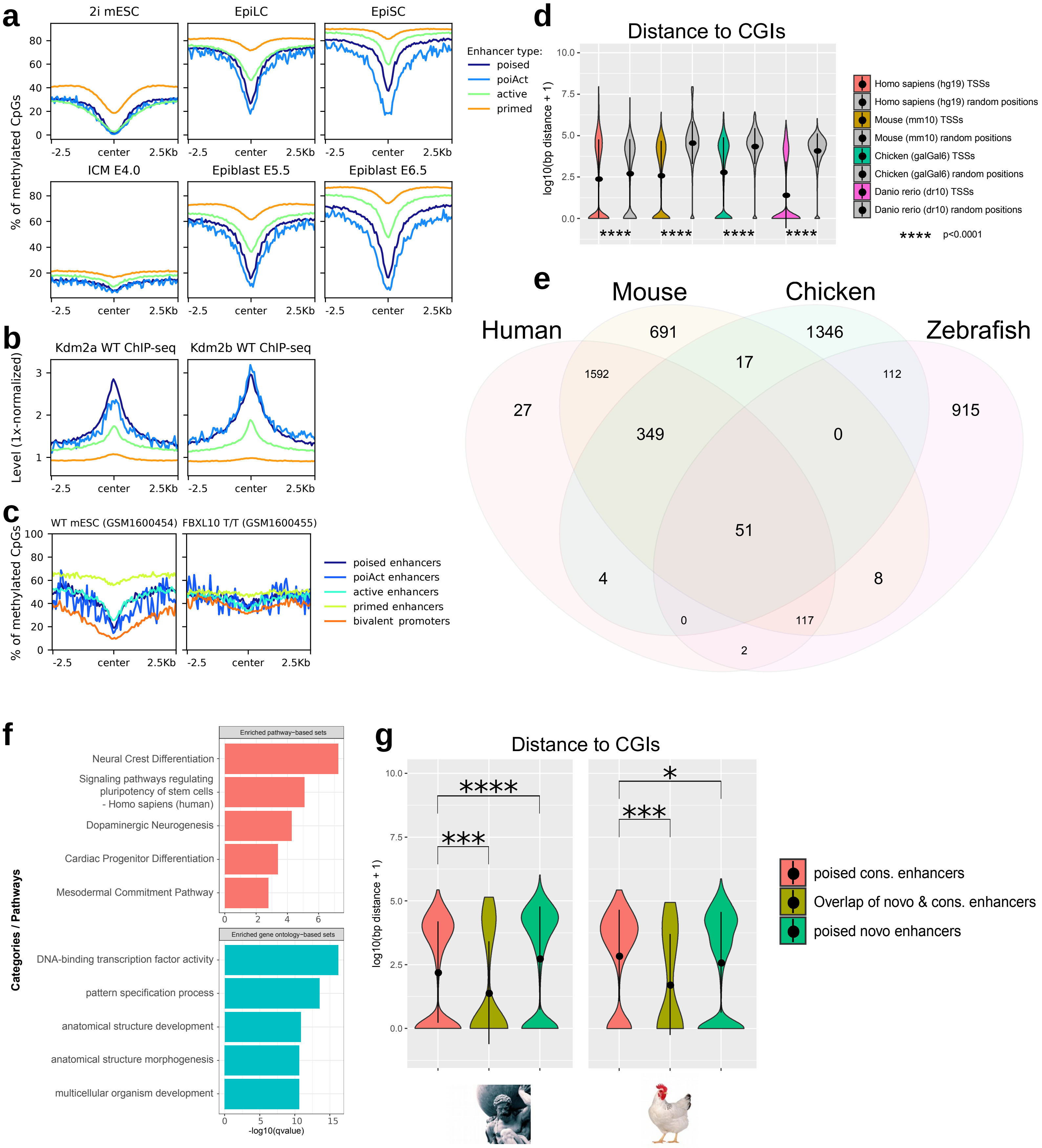
a
2i mESC
EpiLC
EpiSC
ICM E4.0
Epiblast E5.5
Epiblast E6.5
% of methylated CpGs
-2.5
center
2.5Kb
Enhancer type:
poised
poiAct
active
primed
b
Kdm2a WT ChIP-seq
Kdm2b WT ChIP-seq
Level (1x-normalized)
c
WT mESC (GSM1600454)
FBXL10 T/T (GSM1600455)
% of methylated CpGs
poised enhancers
poiAct enhancers
active enhancers
primed enhancers
bivalent promoters
d
Distance to CGIs
log10(bp distance + 1)
Homo sapiens (hg19) TSSs
Homo sapiens (hg19) random positions
Mouse (mm10) TSSs
Mouse (mm10) random positions
Chicken (galGal6) TSSs
Chicken (galGal6) random positions
Danio rerio (dr10) TSSs
Danio rerio (dr10) random positions
**** p<0.0001
e
Human
Mouse
Chicken
Zebrafish
27
691
1346
915
1592
17
112
349
0
51
4
8
0
117
2
f
Enriched pathway-based sets
Neural Crest Differentiation
Signaling pathways regulating pluripotency of stem cells - Homo sapiens (human)
Dopaminergic Neurogenesis
Cardiac Progenitor Differentiation
Mesodermal Commitment Pathway
Enriched gene ontology-based sets
DNA-binding transcription factor activity
pattern specification process
anatomical structure development
anatomical structure morphogenesis
multicellular organism development
Categories / Pathways
-log10(qvalue)
g
Distance to CGIs
log10(bp distance + 1)
poised cons. enhancers
Overlap of novo & cons. enhancers
poised novo enhancers

Supplementary Figure 3: Genetic, epigenetic and regulatory features of PEs are conserved across vertebrates. (A) Whole-genome bisulfite sequencing (WGBS) / STEM-seq data generated in either *in vitro* (2i ESC, EpiLC and EpiSC; upper row) or *in vivo* (E4.0 ICM, E5.5 epiblast and E6.5 epiblast; lower row) mouse pluripotent cells[5-7] was analysed in order to show CpG methylation levels around poised, active, primed and PoiAct enhancers identified in *in vitro* pluripotent cells (**Fig. 1; Methods**). **(B)** ChIP-seq signals for KDM2A and KDM2B in mESC are shown around poised, active, primed and PoiAct enhancers identified in *in vitro* pluripotent cells. **(C)** CpG methylation levels as measured by Reduced-Representation Bisulfite Sequencing (RRBS) in WT mESC and *Kdm2b*$^{-/-}$ mESC[8] are shown around bivalent promoters and poised, active, primed and PoiAct enhancers identified in *in vitro* pluripotent cells. CpG methylation levels increased significantly in *Kdm2b*$^{-/-}$ mESC compared to WT mESC at PE (FC=2.5; p<2.2e-16; two-sided Wilcoxon test), bivalent promoters (FC=3.59; p<2.2e-16; two-sided Wilcoxon test; +/-1kb of TSS) and, to a lesser degree, at active enhancers (FC=1.34; p=3.29e-72; two-sided Wilcoxon test). **(D)** The quality of the CGIs identified by Bio-CAP in different vertebrates is illustrated by the significantly shorter distances separating the CGIs from TSS than from 1000 randomly-sampled genomic regions in each of the investigated species (p<2.2e-16 for all four comparisons; two-sided Wilcoxon test)**. (E)** All the *de novo* PEs identified in different vertebrate species (including the mouse PEs described in **Fig. 1** and located >10kb from TSS) were linked to the nearest gene. Then, homologous genes were retrieved from biomaRt[9] and overlaps between species were calculated with Venn diagrams. **(F)** Pathway and Gene Ontology analysis for the overlapping genes (n=63) shown in (E) were calculated with ConsensusPathDB. **(G)** Distances to CGIs are shown for different PE categories identified in either humans (left panel; p=0.00012; p<2.2e-16 respectively; two-sided Wilcoxon test) or chicken (right panel; p=0.00083; p=0.04998 respectively; two-sided Wilcoxon test). The *de novo* PE overlapping with conserved mouse PE (human: n=183, chicken: n=49) were the most proximal to CGIs.

* p ≤ 0.05, ** p ≤ 0.01, *** p ≤ 0.001, **** p ≤ 0.0001.

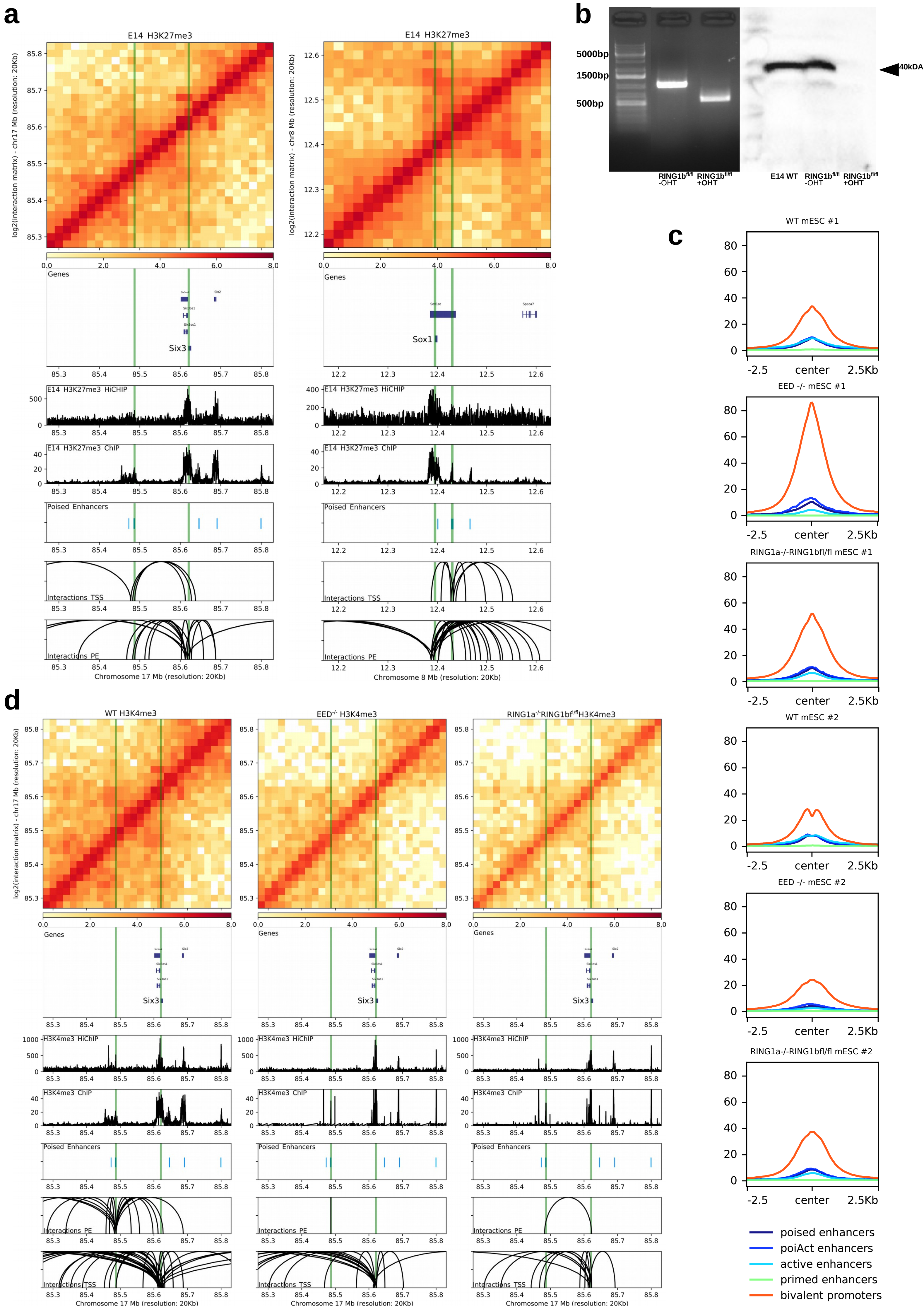

a
E14 H3K27me3
log2(interaction matrix) - chr17 Mb (resolution: 20Kb)
log2(interaction matrix) - chr8 Mb (resolution: 20Kb)
Genes
Six3
Sox1
E14 H3K27me3 HiCHIP
E14 H3K27me3 ChIP
Poised Enhancers
Interactions TSS
Interactions PE
Chromosome 17 Mb (resolution: 20Kb)
Chromosome 8 Mb (resolution: 20Kb)
b
5000bp
1500bp
500bp
RING1b fl/fl -OHT
RING1b fl/fl +OHT
E14 WT
40kDA
c
WT mESC #1
EED -/- mESC #1
RING1a-/-RING1bfl/fl mESC #1
WT mESC #2
EED -/- mESC #2
RING1a-/-RING1bfl/fl mESC #2
-2.5
center
2.5Kb
poised enhancers
poiAct enhancers
active enhancers
primed enhancers
bivalent promoters
d
WT H3K4me3
EED-/- H3K4me3
RING1a-/-RING1bfl/fl H3K4me3
H3K4me3 HiChIP
H3K4me3 ChIP
Chromosome 17 Mb (resolution: 20Kb)

Supplementary Figure 4: The interactions between PEs and bivalent promoters are globally mediated by PRC1. (A) H3K27me3 HiChIP and ChIP-seq profiles generated in mESC are shown around the *Six3* (left) and *Sox1* (right) loci. Both the *Six3 and Sox1* TSS as well as the PEs previously shown to control these genes in AntNPC (PE *Six3* (-133kb) and PE *Sox1* (+35kb)) are marked with green vertical lines. Upper panels: heatmap of log2 interaction intensities based on H3K27me3 HiChIP data generated in mESC. Two medium panels: ChIP-seq and 1D HiChIP signals for H3K27me3 in mESC. Lower two panels: significant ($p<0.01$; FitHiChIP) interactions were called using the H3K27me3 HiChIP PETs (paired-end tags) and loops in which one of the anchors overlaps (+/- 10kb) either the PE or the TSS are shown. **(B)** *Ring1a*$^{-/-}$*Ring1b*$^{fl/fl}$ mESC were either left untreated or treated with tamoxifen for 72 hours. The loss of Ring1b after tamoxifen treatment was confirmed by PCR genotyping (left) in three independent biological replicate experiments and Western blot analysis (right). The PCR primers used for detecting the *Ring1b* deletion are shown in **Supplementary Data 1**. bp=base pairs. **(C)** ChIP-seq data for H3K4me3 was generated as biological replicates in WT mESC, *Eed*$^{-/-}$ mESC and Tamoxifen-treated *Ring1a*$^{-/-}$*Ring1b*$^{fl/fl}$ mESC. H3K4me3 profiles are shown around bivalent promoters and poised, active, primed and PoiAct enhancers identified in *in vitro* mouse pluripotent cells. **(D)** H3K4me3 HiChIP in WT, *Eed*$^{-/-}$ mESC and Tamoxifen-treated *Ring1a*$^{-/-}$*Ring1b*$^{fl/fl}$ mESC around the *Six3* locus.

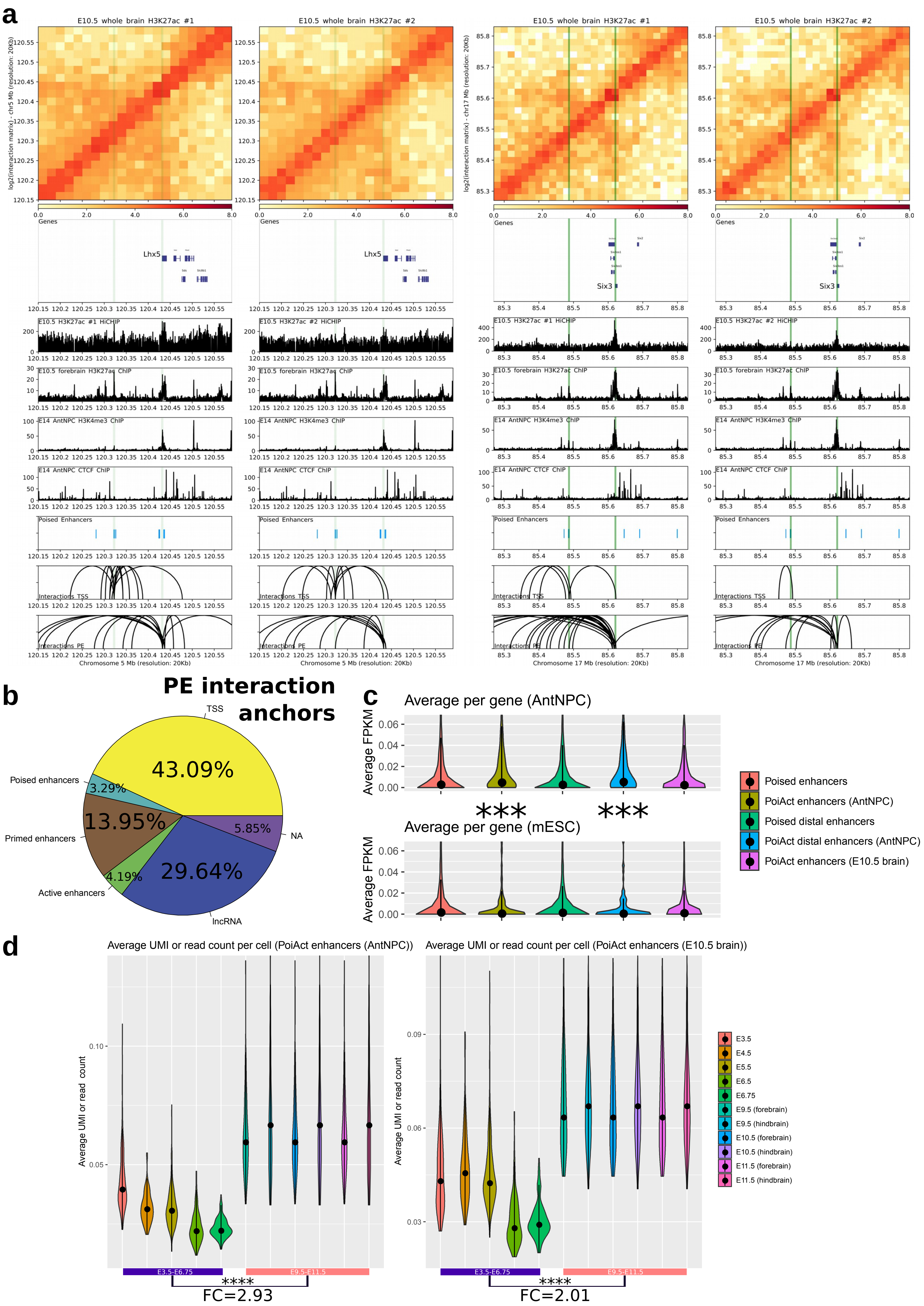

a
E10.5 whole brain H3K27ac #1
E10.5 whole brain H3K27ac #2
log2(interaction matrix) - chr5 Mb (resolution: 20Kb)
log2(interaction matrix) - chr17 Mb (resolution: 20Kb)
Genes
Lhx5
Six3
E10.5 H3K27ac #1 HiCHIP
E10.5 H3K27ac #2 HiCHIP
E10.5 forebrain H3K27ac ChIP
E14 AntNPC H3K4me3 ChIP
E14 AntNPC CTCF ChIP
Poised Enhancers
Interactions TSS
Interactions PE
Chromosome 5 Mb (resolution: 20Kb)
Chromosome 17 Mb (resolution: 20Kb)
b
PE interaction anchors
TSS
43.09%
Poised enhancers
3.29%
Primed enhancers
13.95%
Active enhancers
4.19%
lncRNA
29.64%
NA
5.85%
c
Average per gene (AntNPC)
Average per gene (mESC)
Average FPKM
***
***
Poised enhancers
PoiAct enhancers (AntNPC)
Poised distal enhancers
PoiAct distal enhancers (AntNPC)
PoiAct enhancers (E10.5 brain)
d
Average UMI or read count per cell (PoiAct enhancers (AntNPC))
Average UMI or read count per cell (PoiAct enhancers (E10.5 brain))
Average UMI or read count
E3.5-E6.75
E9.5-E11.5
****
FC=2.93
FC=2.01
E3.5
E4.5
E5.5
E6.5
E6.75
E9.5 (forebrain)
E9.5 (hindbrain)
E10.5 (forebrain)
E10.5 (hindbrain)
E11.5 (forebrain)
E11.5 (hindbrain)

Supplementary Figure 5: Physical interactions between PEs and their target genes in the embryonic mouse brain. (A) H3K27ac HiChIP and ChIP-seq profiles generated in E10.5 mouse brains and AntNPC, respectively, are shown around the *Lhx5* (left; window = +/-200kb) and *Six3* (right; window = +/- 250kb) loci. Both the *Six3 and Lhx5* TSS as well as the PEs previously shown to control these genes in AntNPC (PE *Six3* (-133kb) and PE *Lhx5* (-109kb)) are marked with green vertical lines. Upper panels: heatmap of log2 interaction intensities based on H3K27ac HiChIP data generated in E10.5 mouse brain. Two medium panels: 1D HiChIP and ChIP-seq signals for H3K27ac, H3K4me3 and CTCF in E10.5 mouse brain and AntNPC, respectively. Lower two panels: significant ($p<0.01$; FitHiChIP) interactions were called using the H3K27ac HiChIP PETs (paired-end tags) from E10.5 mouse brain and loops in which one of the anchors overlaps (+/-10kb) either the PEs or the TSS are shown. **(B)** Significant ($p<0.01$; FitHiChIP) interactions were called using the H3K27ac HiChIP PETs from E10.5 mouse brain. Interaction anchors were overlapped with PEs and their interaction partners were hierarchically annotated with the categories shown in the pie chart (**Methods**). **(C)** The target genes of different mouse PE subsets were defined using the significant H3K27me3 HiChIP interactions called in mESC (**Fig. 3**). Then the expression of the genes associated with each PE category was plotted using RNA-seq data generated in AntNPC and mESC. Each data point is the expression (in FPKM) of a target gene. The asterisks indicate significantly higher expression in AntNPC than in ESC (FC=1.33, p=0.00017; FC=1.25, p=0.00035; two-sided Wilcoxon test). The *PoiAct enhancers (E10.5 brain)* category was defined by overlapping mouse PEs with H3K27ac ChIP-Seq peaks (broad; $q \leq 0.1$) called in E10.5 mouse fore-, mid- and hindbrain (**Supplementary Data 1**). Value range was zoomed in to lower and upper IQR (interquartile range) to circumvent excessive outliers. **(D)** The expression of the genes associated with the *PoiAct distal enhancers (AntNPC)* (left) and *PoiAct enhancers (E10.5 brain)* (right) groups described in (C) was plotted using scRNA-Seq data generated during mouse embryogenesis. Each data point is the mean expression (in RPM (reads per million) for E3.5-E6.75 and TPM (transcripts per million) for E9.5-E11.5) of all target genes in one cell. The asterisks indicate significantly higher expression in E9.5-E11.5 brain than in E3.5-E6.75 embryos (FC=2.93, p=3.64e-140; FC=2.01, p=3.41e-124; two-sided Wilcoxon test). Value range was zoomed in to lower and upper IQR to circumvent excessive outliers.

* $p \leq 0.05$, ** $p \leq 0.01$, *** $p \leq 0.001$, **** $p \leq 0.0001$.

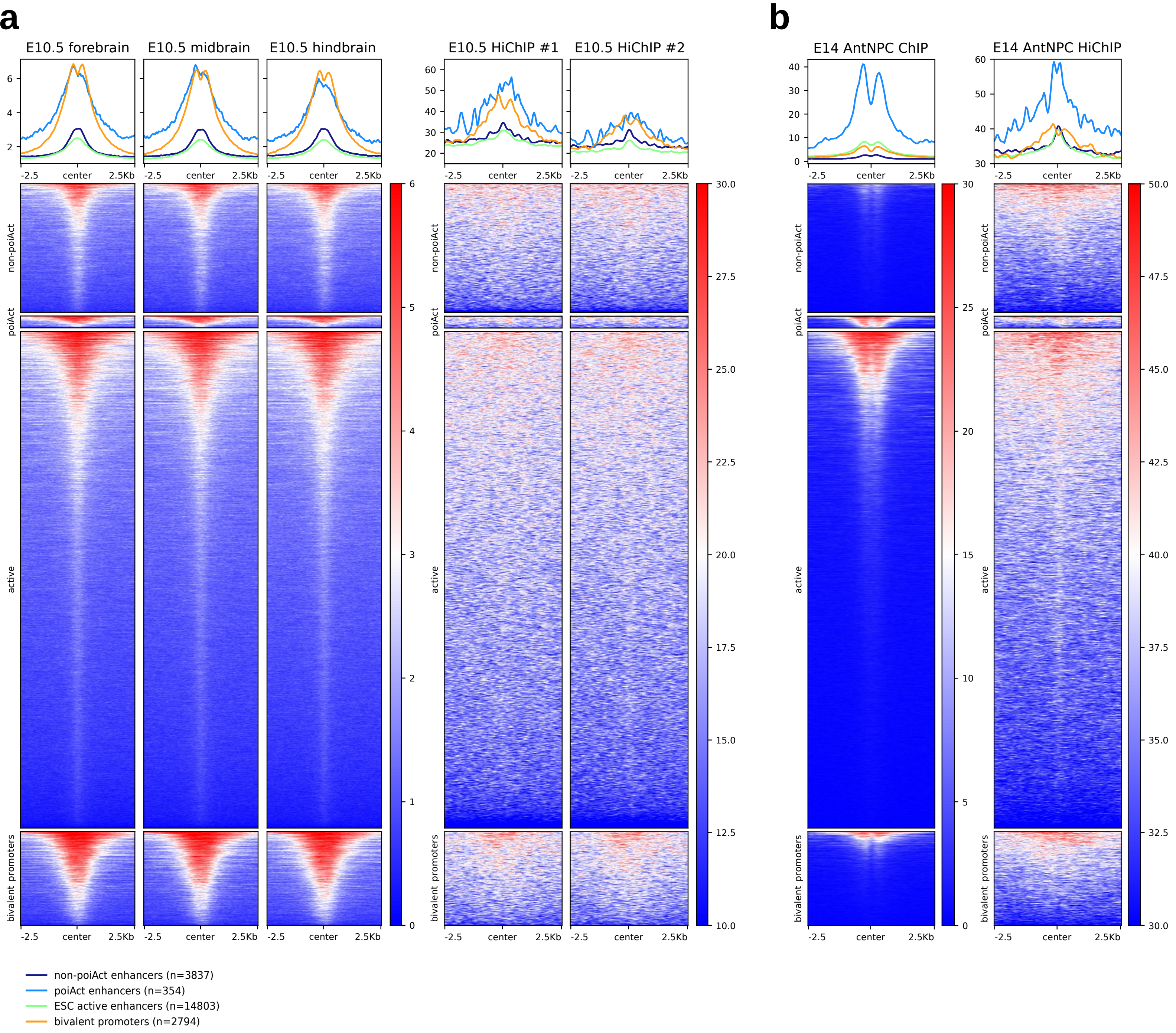
a
E10.5 forebrain
E10.5 midbrain
E10.5 hindbrain
E10.5 HiChIP #1
E10.5 HiChIP #2
b
E14 AntNPC ChIP
E14 AntNPC HiChIP
-2.5
center
2.5Kb
non-poiAct
poiAct
active
bivalent promoters
non-poiAct enhancers (n=3837)
poiAct enhancers (n=354)
ESC active enhancers (n=14803)
bivalent promoters (n=2794)

Supplementary Figure 6: H3K27ac HiChIPs in embryonic brain tissues and differentiated cell lines show specific signal of activated PEs above *in situ* Hi-C background. (A) Public available ENCODE H3K27ac ChIP-seq data (left panel) of the three main brain regions (each with two merged replicates; 1x-normalized) matching the stage of our whole brain HiChIP (right panel) were evaluated in their signal intensity for different regulatory regions. PoiAct enhancers (overlapping H3K27ac peaks in AntNPC), as well as their potential targets in bivalent promoters, show increased read depth compared to residual PEs (non-PoiAct). **(B)** Previously generated E14 AntNPC H3K27ac ChIP-seq data[5] (left panel) and our HiChIP data (right panel) were evaluated in their signal intensity for different regulatory regions. PoiAct enhancers (overlapping H3K27ac peaks in AntNPC), but not as much bivalent promoters compared to **(A)**, show increased read depth compared to residual PEs.

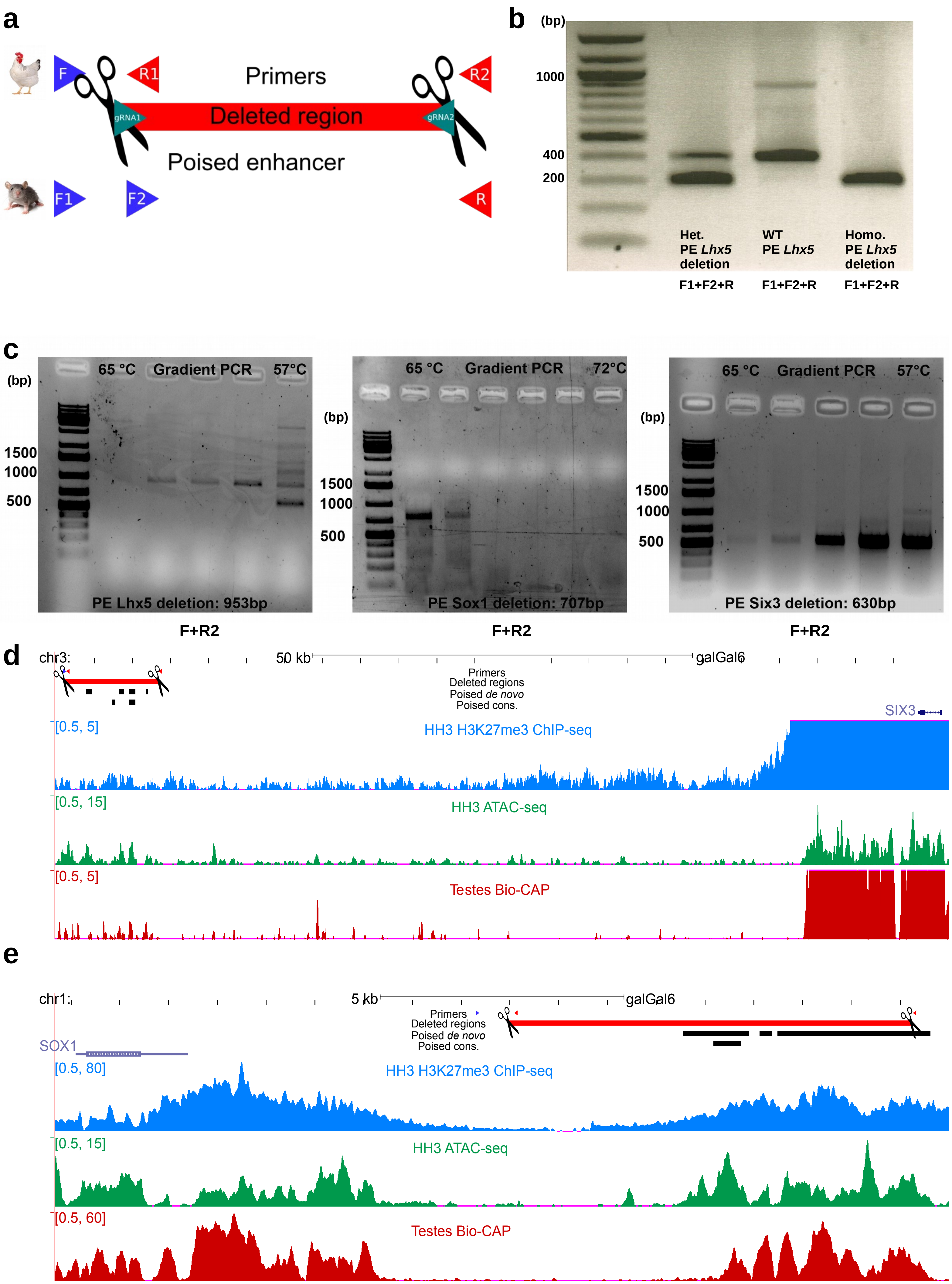
a
F
R1
R2
Primers
gRNA1
Deleted region
gRNA2
Poised enhancer
F1
F2
R
b
(bp)
1000
400
200
Het. PE Lhx5 deletion
WT PE Lhx5
Homo. PE Lhx5 deletion
F1+F2+R
F1+F2+R
F1+F2+R
c
65 °C Gradient PCR 57°C
(bp)
1500
1000
500
PE Lhx5 deletion: 953bp
F+R2
65 °C Gradient PCR 72°C
(bp)
1500
1000
500
PE Sox1 deletion: 707bp
F+R2
65 °C Gradient PCR 57°C
(bp)
1500
1000
500
PE Six3 deletion: 630bp
F+R2
d
chr3:
50 kb
galGal6
Primers
Deleted regions
Poised de novo
Poised cons.
SIX3
[0.5, 5]
HH3 H3K27me3 ChIP-seq
[0.5, 15]
HH3 ATAC-seq
[0.5, 5]
Testes Bio-CAP
e
chr1:
5 kb
galGal6
Primers
Deleted regions
Poised de novo
Poised cons.
SOX1
[0.5, 80]
HH3 H3K27me3 ChIP-seq
[0.5, 15]
HH3 ATAC-seq
[0.5, 60]
Testes Bio-CAP

Supplementary Figure 7: PE deletions in mouse and chicken embryos. (A) Scheme for genotyping of chicken embryos and transgenic mice obtained through CRISPR-Cas9 targeting of PEs. We used one or two forward primers (blue) and one or two reverse primers (red; **Supplementary Data 1**) specific for the deleted and unperturbed region respectively. Primer positions for chicken are further depicted as tracks in **D**, **E** and **Fig. 6E**. **(B)** Genotyping results for the *PE Lhx5(−109kb)* deletion in transgenic mice. Used primer combinations are indicated below the gel. **bp=base pairs. (C)** The CRISPR/Cas9-targeted PEs for *Lhx5*, *Six3* and *Sox1* were PCR-amplified from electroporated chicken genomic DNA using primers specific for the deleted regions. Used primer combinations are indicated below the gel. **(D)** ATAC-seq and H3K27me3 ChIP-seq profiles generated in HH3 chicken epiblast are shown around the *Six3* locus. In addition, Bio-CAP profiles generated in chicken testes are also shown to illustrate the location of CGIs in the chicken genome. The genomic region deleted in chicken embryos that includes the mouse PE *Six3* (-133kb) conserved in chicken is highlighted in red. **(E)** Similar profiles as in (E), but for the mouse PE *Sox1* (+35kb) conserved in chicken.

Supplementary References

1. Bleckwehl, T. et al. Enhancer priming by H3K4 methylation safeguards germline competence. *bioRxiv*. (2020).
2. Zheng, H. et al. Resetting Epigenetic Memory by Reprogramming of Histone Modifications in Mammals. *Mol Cell*. **63**(6):1066-79 (2016).
3. Cruz-Molina, S. et al. PRC2 Facilitates the Regulatory Topology Required for Poised Enhancer Function during Pluripotent Stem Cell Differentiation. *Cell Stem Cell*. **20**(5):689-705.e9 (2017).
4. Bejerano, G. et al. Ultraconserved elements in the human genome. *Science*. **304**(5675):1321-5 (2004).
5. Habibi, E. et al. Whole-genome bisulfite sequencing of two distinct interconvertible DNA methylomes of mouse embryonic stem cells. *Cell Stem Cell*. **13**(3):360-9 (2013).
6. Zylicz, J.J. et al. Chromatin dynamics and the role of G9a in gene regulation and enhancer silencing during early mouse development. *Elife*. **4**:e09571 (2015).
7. Zhang, Y. et al. Dynamic epigenomic landscapes during early lineage specification in mouse embryos. *Nat Genet*. **50**(1):96-105 (2018).
8. Boulard, M., Edwards, J.R. & Bestor, T.H. FBXL10 protects Polycomb-bound genes from hypermethylation. *Nat Genet*. **47**(5):479-85 (2015).
9. Durinck, S., Spellman, P.T., Birney, E. & Huber W. Mapping identifiers for the integration of genomic datasets with the R/Bioconductor package biomaRt. *Nat Protoc*. **4**(8):1184-91 (2009).
