# Supplementary material for: The chromatin, topological and regulatory properties of pluripotency-associated poised enhancers are conserved in vivo
Source: Nat Commun. 2021 Jul 16;12:4344. doi: 10.1038/s41467-021-24641-4 (PMC8285398; doi:10.1038/s41467-021-24641-4)
Supplement: Supplementary file 2 — Description of Additional Supplementary Files [file 41467_2021_24641_MOESM2_ESM.docx]

**Description of Additional Supplementary Files**

File name: Supplementary Data 1

Description: This excel file contains several sheets that provide information about oligonucleotides and computational resources used in our work. Each sheet contains the following information:

*gDNA_primers:* primers used to genotype *Ring1a^-/-^Ring1b^fl/fl^* mESC.

*ISH:* primers used to generate probes for in situ hybridization.

*gRNAs:* oligonucleotides used to generate enhancer deletions using CRISPR/Cas9 technology.

*Genotyping:* primers used to genotype the enhancer deletions generated by CRISPR/Cas9.

*Sessions:* UCSC genome browser sessions used to visualize genomic data in different species.

*Public Data sets:* Accession IDs and publication references for the different genomic data sets used in our work.

*ENCODE (Bing Ren, UCSD):* list of ENCODE genomic data sets used in our study.

*UCSC genomes:* genome assemblies used for the different vertebrate species considered in our work.

*Mapping statistics:* mapping and quality control statistics for the ChIP-seq and HiChIP data generated in this work.

File name: Supplementary Data 2

Description: Genomic coordinates for the different list of enhancers and HiChIP loops considered in our work.
